# Unmasking right paraduodenal hernia: a rare cause of small bowel obstruction

**DOI:** 10.1093/jscr/rjag251

**Published:** 2026-04-09

**Authors:** Anandi Andappan, Rangarajan Govindarajan, Santhoshkumar Selvamani, Rakesh Sharma Kathaiyan

**Affiliations:** Department of General Surgery, Govt Stanley Medical College and Hospital, No.1, Old jail road, Chennai 600 001, Tamil Nadu, India; Department of General Surgery, Govt Stanley Medical College and Hospital, No.1, Old jail road, Chennai 600 001, Tamil Nadu, India; Department of General Surgery, Govt Stanley Medical College and Hospital, No.1, Old jail road, Chennai 600 001, Tamil Nadu, India; Department of General Surgery, Govt Stanley Medical College and Hospital, No.1, Old jail road, Chennai 600 001, Tamil Nadu, India

**Keywords:** internal hernia, right paraduodenal, bowel obstruction

## Abstract

Right paraduodenal hernia (PDH) is one of the congenital internal hernias resulting from developmental error of midgut rotation and is a rare cause of small bowel obstruction. Because of its nonspecific clinical presentation and low index of suspicion, preoperative diagnosis is challenging. We report a case of a 40-year-old male who presented with features of intestinal obstruction. Computed tomography of the abdomen revealed clustered small bowel loops in the right upper quadrant with characteristic displacement of mesenteric vessels, suggestive of a right PDH. Emergency laparotomy confirmed herniation of jejunal and ileal loops through the fossa of Waldeyer with evidence of bowel gangrene. The surgical management followed in our patient is reduction of the herniated bowel, resection of nonviable bowel segments, temporary ostomy, and definitive closure of the hernia defect. The postoperative course was uneventful and the patient recovered well.

## Introduction

An internal hernia is defined as protrusion of a segment of small intestine through a mesenteric or peritoneal defect within the abdominal cavity. It occurs in approximately 0.5%–5.8% of cases presenting with acute small bowel obstruction. Although most internal hernias are congenital, they may also develop following trauma, inflammation, or previous abdominal surgery [1, 2]. Paraduodenal hernia is the most common subtype, representing nearly 50% of all internal hernias. It may occur on either left or right side, the latter being less frequent. In many instances, the diagnosis is made intraoperatively or at autopsy, as preoperative identification remains challenging due to nonspecific clinical presentation. Symptoms may range from vague abdominal pain in cases of spontaneous reduction to severe colicky pain in cases of obstruction [1, 3, 4]. Early diagnosis and prompt surgical intervention are essential to prevent bowel ischemia, as reported with mortality rates of 50% in complicated cases. We report a case of complicated right paraduodenal hernia in a 40-year-old male presented with acute abdomen.

## Case presentation

**Figure 1:**
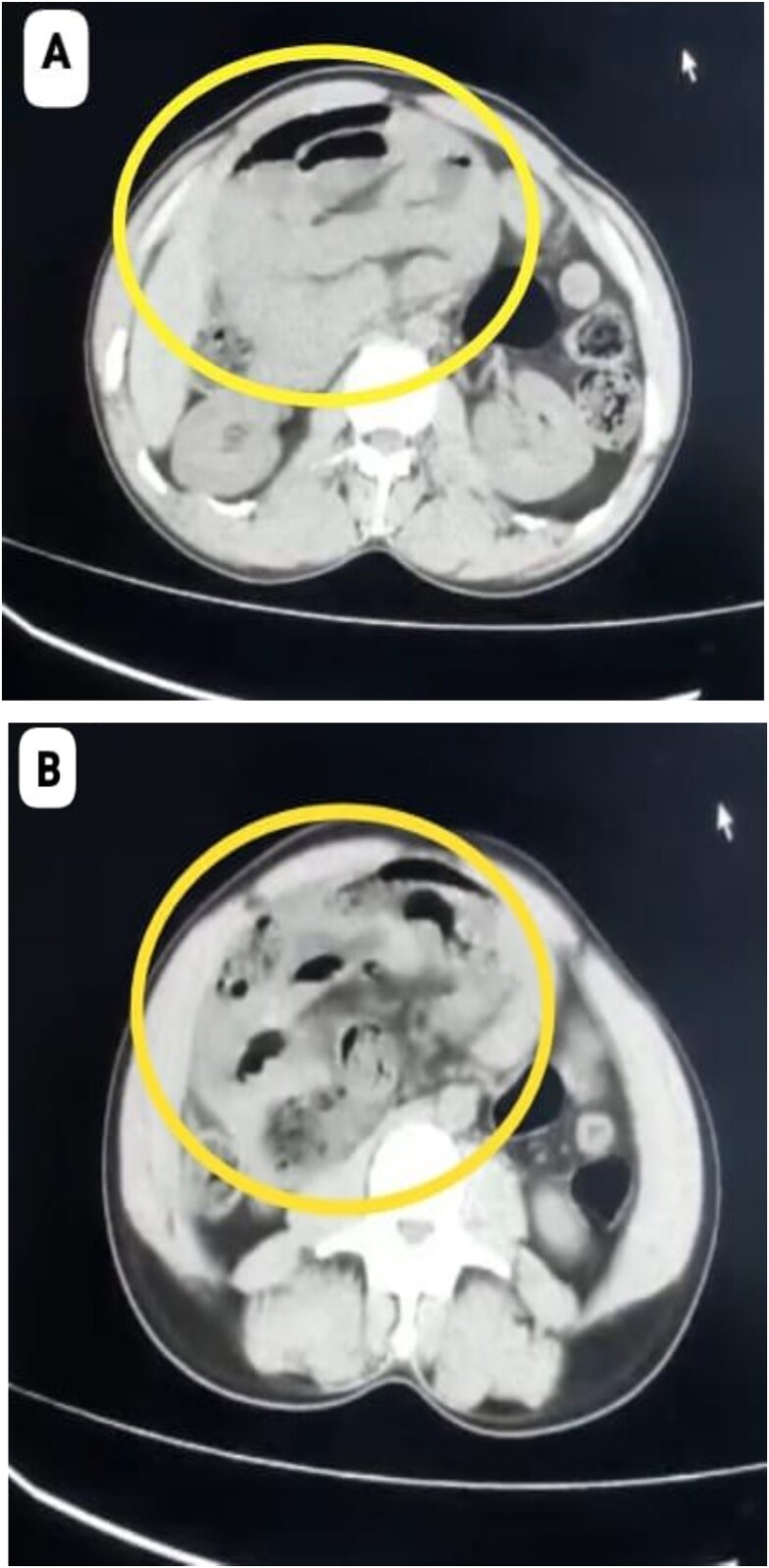
(A, B) CT Showing cluster of small bowel loops in right upper quadrant of abdomen consistent with right paraduodenal hernia.

A 40-year-old male with no known comorbidities and no prior abdominal surgery presented with abdominal pain for 3 days, abdominal distention and vomiting for 1 day, along with obstipation. On admission, he was haemodynamically unstable with a blood pressure of 80/60 mmHg, pulse rate of 124 beats/min, SpO2–96% on room air. Abdominal examination revealed marked distension, diffuse tenderness with guarding and absent bowel sounds. Plain abdominal radiography demonstrated multiple dilated bowel loops with air–fluid levels, predominantly on the right side. Abdominal computed tomography (CT) revealed encapsulated clusters of jejunal and ileal loops inferior to the third part of duodenum with small bowel faeces sign. The superior mesenteric artery (SMA) and superior mesenteric vein (SMV) were seen at the neck of the sac and displaced towards left lateral side, findings consistent with right PDH causing small bowel obstruction (Fig. 1A and B).

**Figure 2:**
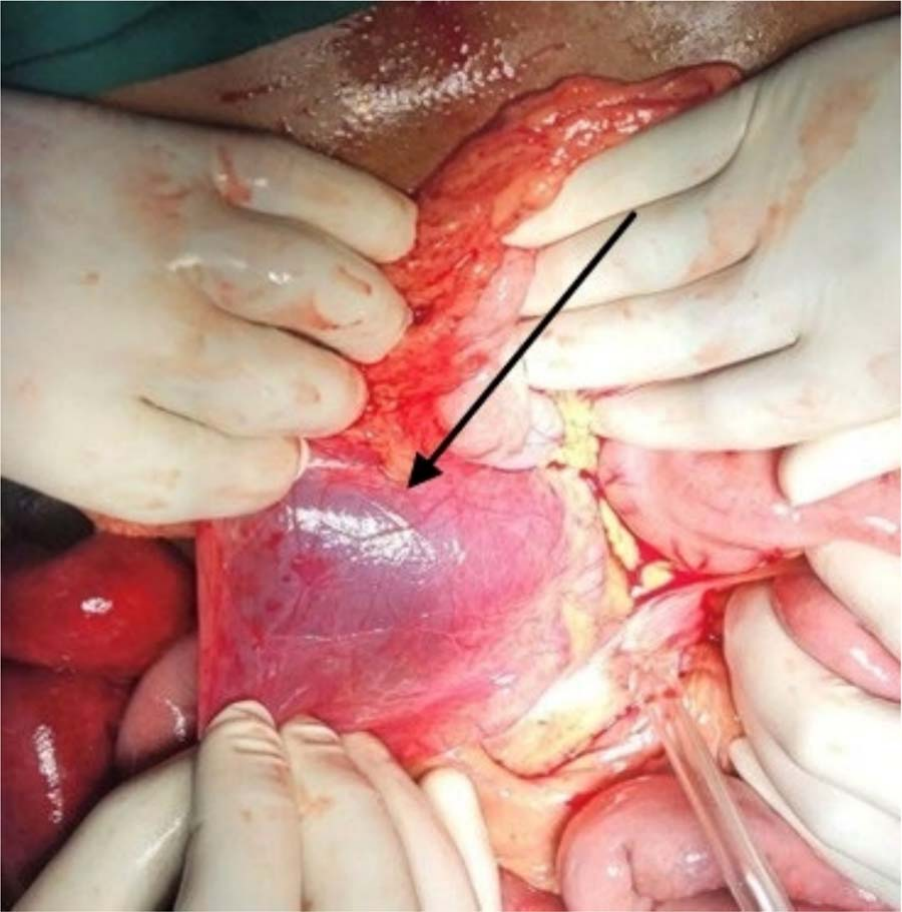
Intraoperative picture showing hernial sac with bowel loops after reflecting ascending colon.

**Figure 3:**
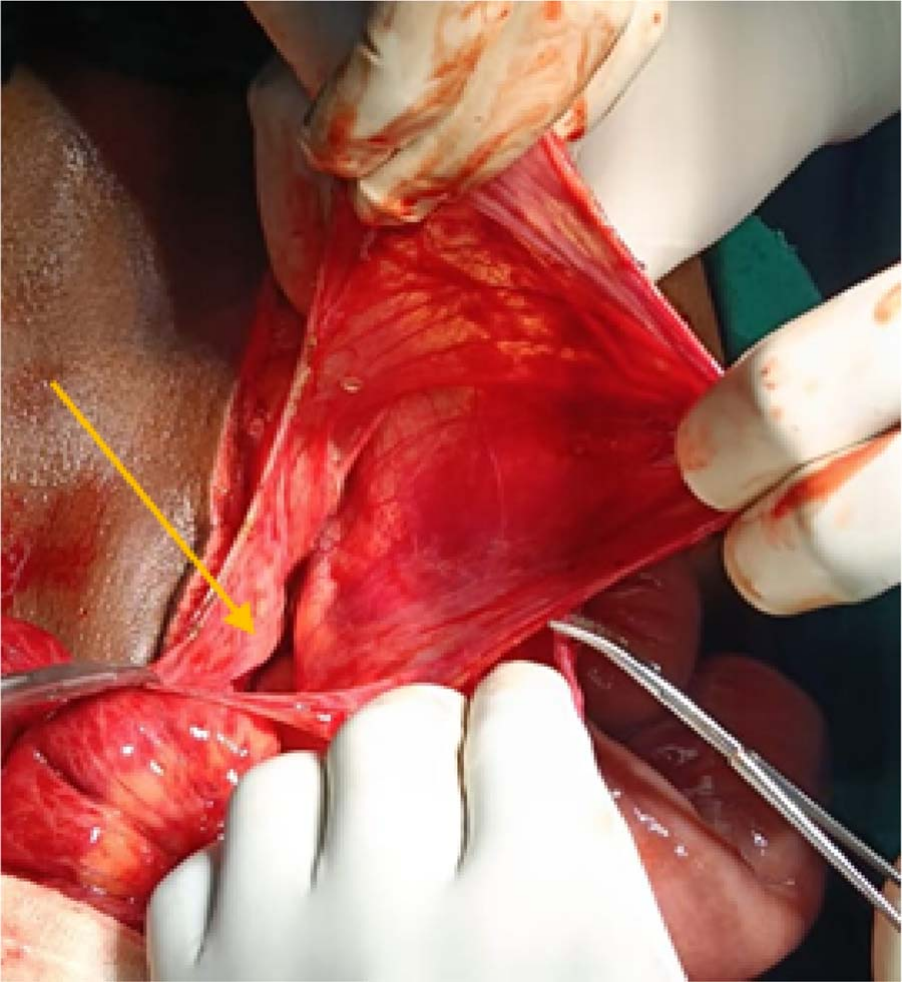
Intraoperative picture showing hernial sac with defect following reduction of bowel loops.

**Figure 4:**
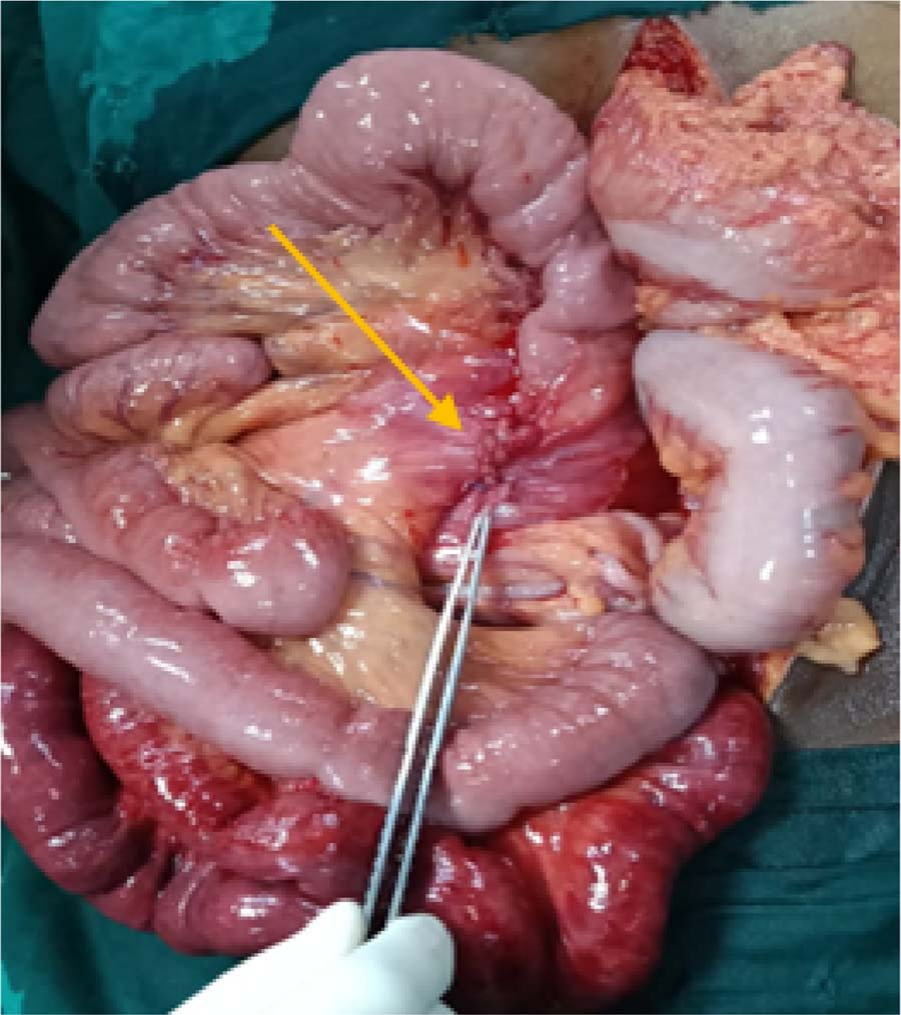
Intraoperative picture showing closure of the defect with gangrenous small bowel.

After initial resuscitation, the patient underwent emergency exploratory laparotomy. Intra-operatively, a large hernial sac was found near the third part of the duodenum behind ascending colon and right side of transverse colon containing gangrenous small bowel loops (Fig. 2). Lateral peritoneal attachments were divided and the caecum, ascending colon were mobilized medially representing the nonrotated gut. This manoeuvre exposed the underlying mesenteric defect. The hernial sac was opened widely which contained ileum and jejunum. After gentle reduction of the contents, the hernial opening was identified as a congenital defect adjacent to the third part of the duodenum, with the superior mesenteric vessels forming its medial boundary (Fig. 3). The margins of the defect within the mesocolon were carefully delineated and closed using absorbable 3-0 Vicryl in a continuous fashion (Fig. 4). Approximately 1 metre of gangrenous ileum, located 30 cm proximal to the ileocaecal junction, was resected. Given the patient’s hemodynamic instability and bowel condition, the proximal ileum was exteriorized as an end ileostomy in the left iliac fossa, and a distal mucus fistula was created in the right iliac fossa. The patient recovered well and was discharged on postoperative day 10.

## Discussion

PDH refers to entrapment of small intestine in a peritoneal sac situated near the third or fourth part of the duodenum. Approximately 10 peritoneal fossae have been described in the literature [1, 5]. Around 75% occur on the left in fossa of Landzert, whereas right paraduodenal hernias account for ~25% and occur through fossa of Waldeyer.

Right PDH arises from abnormal midgut rotation. The prearterial limb fails to undergo 180° counterclockwise rotation during the second stage, while the postarterial segment rotates appropriately to the right. Subsequent fixation of the colon to the posterior peritoneum leads to entrapment of the small bowel behind the mesentery of cecum, ascending and transverse colon [1, 2, 4]. The terminal ileum exits the sac through a small opening to connect with the cecum.

Despite its congenital nature, patients usually present in the third or fourth decades of life with average age of 38.5 years and a male predominance of 3:1 [2]. The most common presentation is small bowel obstruction with colicky abdominal pain, vomiting, distension while others remain asymptomatic as bowel within the sac freely communicate with peritoneal cavity. Some may have experienced indistinct postprandial pain, improved with supine position in the past due to partial obstruction. Flank pain also noted in some patients because of retroperitoneal mass effect [6]. In large hernias, a palpable abdominal mass may occasionally be present.

Preoperative diagnosis is often difficult owing to their nonspecific findings. CT scan remains the investigation of choice in both elective and emergency settings which will show clustered small bowel loops near third part of duodenum with displacement of the SMA, jejunal branches of the SMA and SMV looping posteriorly and to the right. Other findings include SMA, ileocolic artery, and right colic vein in the anterior margin of hernial neck with features of obstruction such as bowel dilatation, mesenteric congestion, and fat stranding [2, 4].

Surgical management is indicated due to the significant 50% lifetime risk of incarceration. The operative principles include reduction of the herniated bowel, identification of the defect in relation to major vascular structures, resection of nonviable segments when required, and closure of the defect to prevent recurrence. The goal is to restore pre and postarterial segments to their normal positions at the end of first stage of rotation, the duodenum, jejunum and most of the ileum to the right, and cecum and colon on the left [7, 8]. Care must be taken whilst addressing the neck of the sac to avoid vascular injury. Excising the sac may reduce the risk of future adhesive obstruction. Depending on the patient condition, decision of performing primary anastomosis or comeback for a second surgery should be made [9]. In patients without bowel necrosis and mild to moderate dilatation, laparoscopic surgery can also be tried [4, 7, 10].

## Conclusion

Right PDH is a rare but potentially life-threatening cause of intestinal obstruction particularly in patients without prior abdominal surgery. Its nonspecific clinical presentation often delays diagnosis, increasing the risk of bowel gangrene and associated septic complications. CT plays a crucial role in early identification. A thorough understanding of the relevant embryological and vascular anatomy is essential for safe surgical management and to minimize the risk of vascular injury.
